# Supplementary material for: A Critical View of the Application of the APEX Software (Aqueous Photochemistry of Environmentally-Occurring Xenobiotics) to Predict Photoreaction Kinetics in Surface Freshwaters
Source: Molecules. 2019 Dec 18;25(1):9. doi: 10.3390/molecules25010009 (PMC7017383; doi:10.3390/molecules25010009)
Supplement: Supplementary file 1 [file molecules-25-00009-s001.zip › 3.2.4_gcc-4.4.0.zip!/3.2.4_gcc-4.4.0/3.2.4_gcc-4.4.0/doc/octave/html/interpreter/Printing-Notation.html]

Printing Notation - Untitled


Next: Error Messages,
Previous: Evaluation Notation,
Up: Conventions


---

#### 1.3.3 Printing Notation

Many of the examples in this manual print text when they are
evaluated. In this manual the printed text resulting from an example
is indicated by -|. The value that is returned by
evaluating the expression is displayed with =>
(`1` in the next example) and follows on a separate line.

```
     printf ("foo %s\n", "bar")
          -| foo bar
          => 1
```
